# Multi-walled carbon nanotubes wrapped with polyvinylpyrrolidone can control the leaf yellowing of *Alstroemeria* cut flowers

**DOI:** 10.1038/s41598-022-18642-6

**Published:** 2022-08-20

**Authors:** Seyedeh Farzaneh Mousavi, Zeynab Roein, Seyedeh Hoda Hekmatara

**Affiliations:** 1 Department of Horticultural Sciences, Faculty of Agriculture, Ilam University, P.O. Box 69315-516, Ilam, Iran; 2 Department of Physics, Faculty of Science, Vali-e-Asr University of Rafsanjan, Rafsanjan, Iran

**Keywords:** Plant sciences, Plant physiology

## Abstract

The rapid yellowing of the leaves on cut flowers with leafy stems severely limits their vase life and commercial value. In this study, the effect of a composite of multi-walled carbon nanotubes (MWCNTs) and polyvinyl pyrrolidone (PVP) on the longevity of cut *Alstroemeria* flowers (*Alstroemeria hybrida*) was investigated to obtain a solution to this problem. A range of MWCNTs/PVP composite concentrations (0, 3, 6, and 9 mg L^−1^) was applied in a vase solution (for 24 h) as pulse treatments. Our findings indicate that the composite of MWCNTs and PVP exhibits excellent dispersibility in a vase solution. The results demonstrate that a 3 mg L^−1^ MWCNTs/PVP concentration was the most effective, extending the vase life of cut *Alstroemeria* flowers by up to 27 days. Pulsing with MWCNTs/PVP delayed the onset of floret abscission and leaf yellowing by 5 and 18 days, respectively. Additionally, when MWCNTs/PVP solution was applied to cut stems, water uptake remained consistently greater than that of the control. Additionally, MWCNTs/PVP increased the total chlorophyll content, soluble protein content, and POX enzyme activity of leaves while decreasing the malondialdehyde (MDA) content. The results indicate that this composite exhibited antimicrobial activity against gram-positive and -negative bacteria, particularly at a concentration of 3 mg L^−1^. This study demonstrated that adding MWCNTs/PVP to a vase solution of *Alstroemeria* cut flowers increased their longevity with minimal leaf yellowing symptoms compared to untreated cut stems. As a result, this nanocomposite can be used safely and effectively in vase solutions and in combination with other preservatives.

## Introduction

Carbon nanotubes (CNTs) are thin rolled-up sheets of graphene that exhibit sp^2^ hybridization^1^ and are considered the nanomaterials family's prime spotlight^2^. Due to its superior properties, it has demonstrated outstanding performance in various applications^3^. Carbon nanotubes (CNTs) are classified as single-walled carbon nanotubes (SWCNTs) or multi-walled carbon nanotubes (MWCNTs) based on the number of concentric layers of rolled graphene sheets^4,5^. CNTs can interact with plant tissue, enhancing biological activity^6^. Their effect is generally proportional to the size, concentration, and solubility of the nanomaterials used^7^.

Because carbon-based nanomaterials are hydrophobic, insoluble, or have a low dispersive capacity, they aggregate in aqueous solutions or on the surface of tissues^8–11^. However, this property enhances their ability to interact with various organic compounds^12^. Nevertheless, their hydrophobicity is one of the issues preventing CNTs from being used in agriculture and plant science. Consequently, the addition of CNT reinforcement can improve the mechanical, electrical, and thermal properties^3^. Numerous methods (both covalent and noncovalent) have been introduced for achieving uniform nanotube dispersions^10^. Surfactants (anionic, cationic, and nonionic) and polymer wrapping are efficient nanotube dispersants^8,13,14^. Polyvinyl pyrrolidone (PVP) is a polymer with a hydrophobic alkyl backbone and hydrophilic pendant groups that can coil around the nanotube, exposing the pyrrolidone groups to water. CNTs can be dissolved in water through a noncovalent functionalization procedure involving PVP^14^.

CNTs have been shown to have various effects on plants, including both positive and negative effects, as well as contradictory effects^15^. Multiple types of engineered nanoparticles have been shown to benefit agricultural plant growth, yield, and quality. CNTs functionalized with carboxyl groups, on the other hand, are more readily assimilated and translocated from the roots to the leaves^16^. Additionally, CNTs exhibit a high level of antimicrobial activity^17^. The vascular system transport CNTs via transpiration through the xylem toward the leaves^15,18^. Previous research has demonstrated that CNTs can improve wheat germination and root elongation^19,20^, increase gram, broccoli, and rice growth as well as water uptake^21–23^, facilitate ionic nutrient uptake in maize and broccoli^24,25^, accelerate the expression of water channel protein in tomato plants^26^, increase flower and leaf number in *Catharanthus*^27^, decrease the time required for Bitter Melon to fructify^28^, improve vascular tissues, and enhance the chlorophyll content and photosynthetic activity in rice^23^. Furthermore, adverse effects of CNTs have also been observed in plants^29,30^.

Cut flowers are an integral part of the floriculture industry that can benefit from nanomaterials' vase-life extending properties. Cut flowers cannot absorb sufficient water even when held in vase water^31^ because air bubbles, microbial growth, and deposition of lignin and other substances obstruct the vascular bundles in the cut stems^32^. The most common method of extending the vase's life is to add preservative chemicals to the vase solution^33^. These chemicals typically aid in the rapid rehydration of cut flowers by increasing water uptake and maintaining cell turgor. Nanomaterials can diffuse into vase water and travel through stem vessels^34^. As a result, using these compounds as a preservative solution can improve the water balance in the stem. There have been numerous reports of increased flower longevity due to the use of various nanomaterials^31,33,35,36^, but there are limited reports and data on the effect of carbon nanotubes on cut flower postharvest.

Leaf senescence has a detrimental effect on the quality and vase life of several cut flowers, including *Lilium*^37–39^, *Chrysanthemum*^40^, *Matthiola incana*^41^, *Alstroemeria*^42^, *Gladiolus*^39^, and *Rosa*^43^. Gibberellins, cytokinins, hydrogen peroxide, salicylic acid, and hot water were frequently used in these studies to prevent leaf yellowing. *Alstroemeria* is a popular cut flower in the international flower market and is a member of the Alstroemeriaceae family^44^, favored for its showy and long-lasting vase life (2 weeks or longer) that exists in various colors and patterns (purple, lavender, red, pink, white, orange, peach, yellow, and bi-colors)^45^. The first symptom of senescence in *Alstroemeria* is yellowing of the leaves; this is an undesirable process that can occur within a few days and progress very quickly^46,47^; thus, it deserves special attention.

In this study, we hypothesized that (i) MWCNTs/PVP composites could help delay leaf yellowing in cut *Alstroemeria* flowers, and (ii) they could help regulate water balance in cut stems. To this end, this study aimed to: (a) characterize postharvest quality, (b) investigate the physiological responses of leaves to the MWCNTs/PVP composite, and (c) assess the antimicrobial activity of MWCNTs/PVP in cut stems.

## Results

### Fourier transform infrared (FTIR) spectroscopy

**Figure 1:**
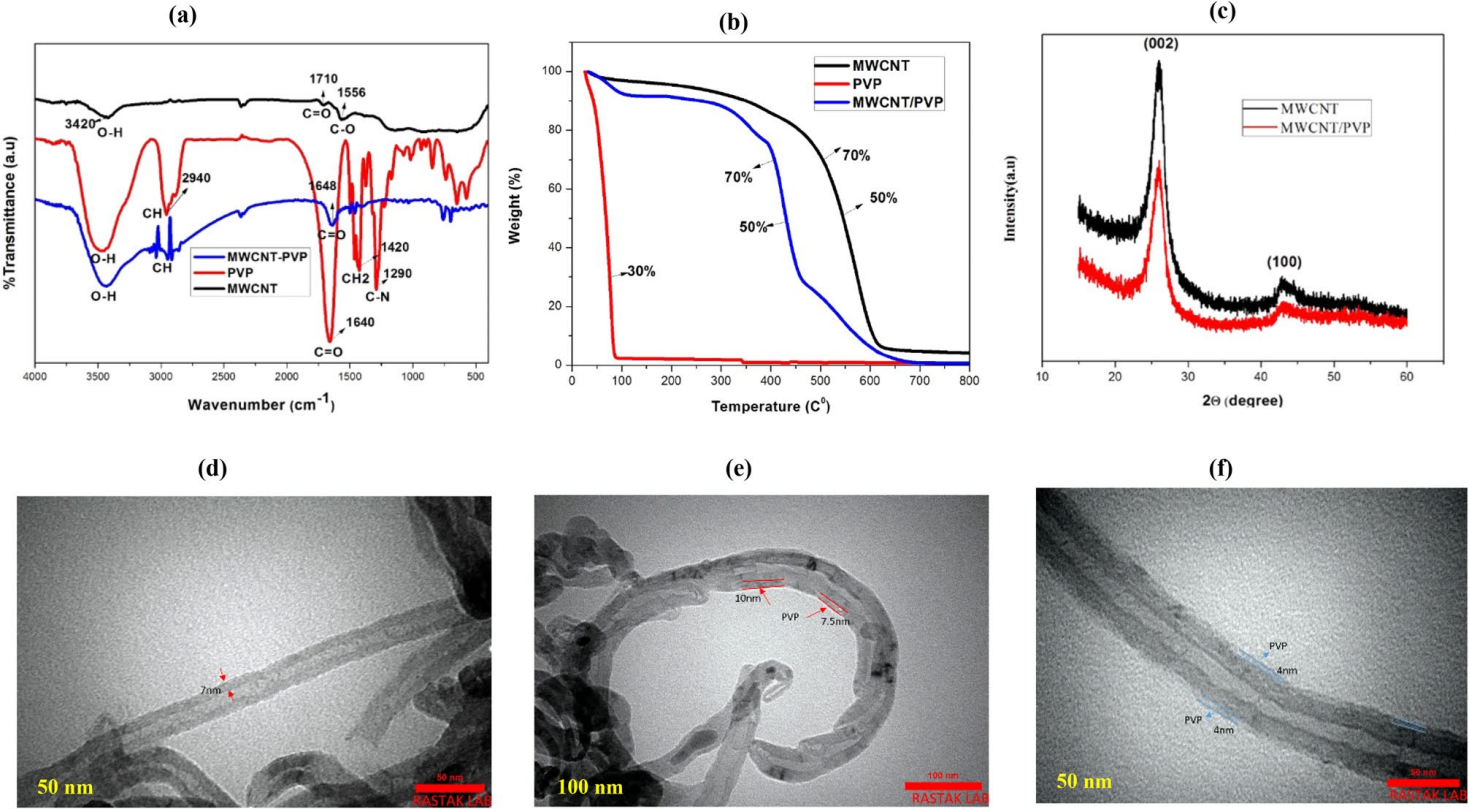
(**a**) FTIR (Fourier-transform infrared spectroscopy) spectra of the pristine MWCNTs (Multi-Walled Carbon Nanotubes), neat PVP (Polyvinylpyrrolidone), and PVP coated MWCNTs. (**b**) TGA (Thermogravimetric analysis) curves of pristine MWCNTs, PVP, and PVP-coated MWCNTs. (**c**) X-ray diffraction patterns of MWCNTs and PVP-coated MWCNTs. (**d**) TEM (Transmission electron microscopy) image of pristine MWCNTs. (**e**) TEM image of PVP-coated MWCNTs at low magnification (scale bar 100 nm). (**f**) TEM image of PVP-coated MWCNTs at high magnification (scale bar 50 nm).

The FTIR spectra of MWCNT, PVP, and MWCNTs/PVP are shown in Fig. 1a.

The FTIR spectra of MWCNT*:* The vibrational mode C=O of the carboxyl group is observed at 1710 cm^−1^, and the tensile band O–H is observed at 3420 cm^−1^, confirming the existence of the carboxyl group COOH in MWCNT. The C–O flexural bond is associated with the carbonyl group of MWCNT. The FTIR spectra of PVP: The C–N tensile band is observed at 1290 cm^−1^, CH_2_ flexural band at 1420 cm^−1^, and CH tensile band at 2940 cm^−1^. The C=O tensile band is observed at 1640 cm^−1^. The O–H tensile band is observed at 3480 cm^−1^.

The FTIR spectra of MWCNTs/PVP: The C=O tensile band is observed at 1648 cm^−1^, and the CH tensile band at 2946 cm^−1^. The O–H tensile band is observed at 3440 cm^−1^. The C=O tensile band of MWCNTs/PVP is at 1648 cm^−1^, exhibiting a red shift compared to C=O of MWCNT (C=O of MWCNT is at 1710 cm^−1^ and C=O of PVP is at 1640 cm^−1^) indicating the formation of a complex between the carboxyl groups of MWCNT and PVP. The CH tensile bond of MWCNTs/PVP appeared with a lower intensity than that of pure PVP, indicating the formation of a bond between MWCNTs and PVP. The absence of C–N and CH_2_ bonds, characteristic of pure PVP in MWCNTs/PVP, reveals the bond between the MWCNTs and PVP (PVP wrapping around the MWCNTs). In MWCNTs/PVP, most PVP bonds appeared with less intensity than pure PVP, demonstrating the lower weight percentage of PVP compared to MWCNTs. This study aimed to place PVP between individual MWCNTs to prevent their accumulation and bundling, thereby enhancing the dispersibility of MWCNTs in an aqueous solvent.

### Thermogravimetric analysis (TGA)

The thermogravimetric curve depicted in Fig. 1b indicates that the polymer (PVP) has a very low thermal stability, as 70% of the polymer decomposes at 77 °C, and the polymer is completely destroyed at 100 °C. The TGA curve of pure MWCNTs shows that the MWCNTs have a very high thermal stability. Only 30% is degraded up to 508 °C, and half of the samples remained over 550 °C. TGA curve of MWCNTs/PVP composite shows that 30% of the sample is degraded at 407 °C, which is 100 °C less than the pure MWCNTs. Therefore, the stability of MWCNTs/PVP composite is approximately 100 °C less than pure MWCNTs. However, MWCNTs/PVP composite demonstrates acceptable thermal stability up to 480 °C. Consequently, MWCNTs significantly improve the thermal stability of PVP.

### X-ray diffraction (XRD) pattern

The X-ray diffraction (XRD) patterns of pure MWCNTs and MWCNTs/PVP are shown in Fig. 1c. The characteristic peaks of the MWCNTs were observed at 2Ɵ = 26° and 43°, corresponding to the (002) and (100) of graphite’s hexagonal structure. The PVP is an amorphous polymer showing no peak in the XRD pattern. Due to X-ray absorption by the polymer layer, the peak intensities of MWCNTs/PVP are lower than those of pure MWCNTs^48^.

### Transmission electron microscopy (TEM)

TEM Images of pure MWCNT and MWCNTs/PVP are depicted in Fig. 1d–f. In the TEM image of pure MWCNT, a multi-walled nanotube with a uniform wall thickness of 12.5 nm is visible. The TEM image of the MWCNTs/PVP reveals that a nearly uniform layer of polymer with a thickness of 7.5–10 nm covers the wall of the MWCNTs, whose wall thickness has reached 20 nm.

### Vase life

Compared to the control treatment, pulsing *Alstroemeria* with all MWCNTs/PVP composite concentrations increased flower longevity (Table 1). MWCNTs/PVP at a concentration of 3 mg L^−1^ increased the vase life of cut flowers to 26.67 days, whereas cut stems held in water lasted only 8.33 days and exhibited quality loss. Vase life was extended by 15.67 days (46.84% extension) and 14.67 days (76.11% extension) at 6 and 9 mg L^−1^ MWCNTs/PVP composite, respectively. Increased nanocomposite concentrations caused a loss of freshness in the leaves following pulsing, and black spots were observed in the cut stem end where MWCNTs aggregated within the xylem.

**Table 1:** Vase life, floret abscission, and leaf yellowing of cut *Alstroemeria* flowers pulsed for 24 h with multi-walled carbon nanotubes (MWCNTs)/polyvinylpyrrolidone (PVP) composite during the vase period.

MWCNTs/PVP (mg L^−1^)	Vase life (d)	Floret abscission (d)	Leaf yellowing (d)
0 (Control)	8.33 ± 0.7 c	8.83 ± 0.1 b	11.89 ± 0.4 c
3	26.67 ± 1.6 a	14.07 ± 0.07 a	30.33 ± 0.8 a
6	15.67 ± 1.2 b	12.15 ± 0.4 a	22.22 ± 1.3 b
9	14.67 ± 0.8 b	12.66 ± 0.6 a	21.33 ± 0.3 b
F-test probability	< 0.0001	< 0.0001	< 0.0001
LSD 0.01 (n = 3)	5.6	1.96	3.6

The mean values followed by the same superscript letter are not significantly different at *P* ≤ 0.05, using LSD test. Values are means ± SE of three replicates.

### Floret abscission

Compared to the control, MWCNTs/PVP composite treatment increased the average floret abscission (Table 1). After 8.33 days, the control florets had already been abscised, whereas cut flowers treated with MWCNTs/PVP (at 3 mg L^−1^) remained intact for an additional 14 days before falling. After 12 days, abscission of the first floret was observed in all of the cut stems and was treated with 6 and 9 mg L^−1^ MWCNTs/PVP.

### Leaf yellowing

**Figure 2:**
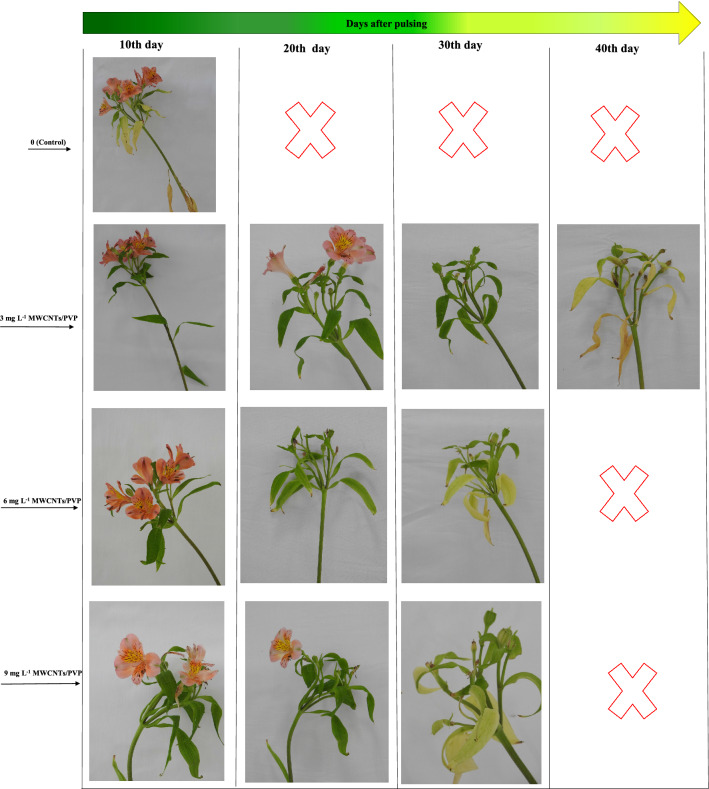
Leaf yellowing condition in cut *Alstroemeria* flowers treated with different concentrations of multi-walled carbon nanotubes (MWCNTs)/polyvinylpyrrolidone (PVP) composite (0, 3, 6, and 9 mg L^−1^).

Compared to the control, all MWCNTs/PVP pulse treatments delayed the yellowing of cut *Alstroemeria* (Table 1). MWCNTs/PVP pulse treatment at a concentration of 3 mg L^−1^ delayed the yellowing of cut *Alstroemeria* leaves. Additionally, this treatment tripled the control's leaf longevity and preserved the leaves green color for 30 days. Before day 12, the entire foliage on control stems appeared severely wilted, and most leaves had yellowed. On day 20, cut stems treated with 6 and 9 mg L^−1^ MWCNTs/PVP remained green. An intriguing facet of this case of *Alstroemeria* leaf yellowing was the variation in leaf yellowing patterns between *Alstroemeria* species. The leaves of nanocomposite-treated plants gradually lost their green color from the bottom of the cut stem upward (and near the florets), whereas all leaves of the cut stem were yellowed when flowers were treated with distilled water (Fig. 2).

### Relative fresh weight

Table 2 illustrates the variation in the fresh weight of cut stems during the vase life. The relative fresh weight decreased gradually except on days 1 and 3, when it increased rapidly, particularly in flowers kept in 3 mg L^−1^ MWCNTs/PVP. The cut stems in this treatment maintained a higher fresh weight than at the start of the experiment. Cut stems treated with 9 mg L^−1^ MWCNTs/PVP exhibited a lower fresh weight than stems treated with 6 mg L^−1^ MWCNTs/PVP. After 5 days, cut stems kept in water (control) began to lose weight and, after 9 days, had a lower fresh weight than their initial value.

**Table 2:** Relative fresh weight of cut *Alstroemeria* flowers pulsed for 24 h with multi-walled carbon nanotubes (MWCNTs)/polyvinylpyrrolidone (PVP) composite during the vase period.

MWCNTs/PVP (mg L^−1^)	Relative Fresh weight (% of initial)						
	Time (d)						
	0	1	3	5	7	9	11
0 (Control)	100 ± 0 g–k	104 ± 0.1e–i	104 ± 2.1e–i	94 ± 1.5 klm	76 ± 2.08 o	64 ± 2.9 p	0.0 q
3	100 ± 0 g–k	113 ± 3.2 abc	118 ± 4 a	116 ± 4.8 ab	110 ± 4.4 b–e	105 ± 0.26 e–i	99 ± 0.33 hi
6	100 ± 0 g–k	107 ± 0.4 c–f	108 ± 2.5 cde	112 ± 0.55 a–d	109 ± 1.3 cde	99 ± 4.3 jkl	93 ± 3.3 lm
9	100 ± 0 g–k	106 ± 0.4 d–g	105 ± 2.2 e–h	101 ± 2.2 f.–j	97 ± 1.9 g–m	92 ± 1.6 m	85 ± 1.1 n
F-test probability	< 0.0001						
LSD 0.05 (n = 3)	6.3						

The mean values followed by the same superscript letter are not significantly different at *P* ≤ 0.05, using LSD test. Values are means ± SE of three replicates.

### Water uptake

In this study, it was observed that water uptake in the vase solution followed a pattern similar to the relative fresh weight of the cut flower, as the water absorption rate initially increased but then gradually decreased with time (Table 3). Among the treatments, 3 mg L^−1^ MWCNTs/PVP had the greatest effect on cut flower stem water uptake. The maximum water uptake was 2.09 and 1.79 L kg^−1^ on days 3 and 5, respectively, and decreased to 1.29 L kg^−1^ on day 11, with the stems in the water (control) exhibiting the least. Cut stems placed in 6 and 9 mg L^−1^ MWCNTs/PVP preservative solutions, on the other hand, absorbed more of the vase solutions than control stems.

**Table 3:** Water uptake of cut *Alstroemeria* flowers pulsed for 24 h with multi-walled carbon nanotubes (MWCNTs)/polyvinylpyrrolidone (PVP) composite during the vase period.

MWCNTs/PVP (mg L^−1^)	Water uptake (L kg^−1^)					
	Time (d)					
	1	3	5	7	9	11
0 (Control)	0.68 ± 0.02 gh	1.61 ± 0.007 c	1.43 ± 0.009 d	0.88 ± 0.02 ef	0.65 ± 0.004 gh	0.0 j
3	0.75 ± 0.02 g	2.09 ± 0.02 a	1.79 ± 0.01 b	1.6 ± 0.01 c	1.36 ± 0.02 d	1.29 ± 0.0 d
6	0.49 ± 0.02 i	0.99 ± 0.02 d	1.5 ± 0.02 i	1.3 ± 0.01 gh	1.1 ± 0.01 g–i	0.97 ± 0.2 hi
9	0.56 ± 0.09 hi	0.7 ± 0.08 e	1.4 ± 0.009 i	1.2 ± 0.02 fg	1 ± 0.03 hi	0.94 ± 0.05 i
F-test probability	< 0.0001					
LSD 0.05 (n = 3)	0.16					

The mean values followed by the same superscript letter are not significantly different at *P* ≤ 0.05, using LSD test. Values are means ± SE of three replicates.

### Chlorophyll content

The total chlorophyll content of leaves decreased during the vase life of control cut stems. MWCNTs/PVP treatments altered the pattern of chlorophyll decline during vase life in a different way from that observed in control stems (Table 4). Over the first 11 days, pulse treatment with 3 mg L^−1^ MWCNTs/PVP resulted in a gradual decline in total chlorophyll. On day 11, the chlorophyll content of leaves at the start of vase life ranged between 0.71 and 0.48 g kg^−1^. During the vase life of the remaining flowers, a decrease in chlorophyll content was observed.

**Table 4:** Total chlorophyll content in leaf of cut *Alstroemeria* flowers pulsed for 24 h with multi-walled carbon nanotubes (MWCNTs)/polyvinylpyrrolidone (PVP) composite during the vase period.

MWCNTs/PVP (mg L^−1^)	Total chlorophyll (g kg^−1^)					
	Time (d)					
	1	3	5	7	9	11
0 (Control)	0.51 ± 0.04 de	0.37 ± 0.01 h–k	0.36 ± 0.009 h–k	0.29 ± 0.003 lm	0.21 ± 0.01 m	0.00 n
3	0.71 ± 0.007 a	0.66 ± 0.01 ab	0.65 ± 0.04 ab	0.60 ± 0.05 bcd	0.50 ± 0.01e	0.48 ± 0.01 ef
6	0.47 ± 0.08 efg	0.46 ± 0.05 e–h	0.45 ± 0.03 e–i	0.45 ± 0.01 e–j	0.37 ± 0.02 h–l	0.34 ± 0.02 kl
9	0.62 ± 0.06 abc	0.52 ± 0.03 cde	0.38 ± 0.01 e–i	0.42 ± 0.03 e–k	0.39 ± 0.02 e–k	0.35 ± 0.01 jkl
F-test probability	< 0.0001					
LSD 0.05 (n = 3)	0.09					

The mean values followed by the same superscript letter are not significantly different at *P* ≤ 0.05, using LSD test. Values are means ± SE of three replicates.

### Leaf soluble protein content

Table 5 illustrates the variations in soluble protein content of cut flowers. MWCNTs/PVP pulsing affected the protein content. Flowers kept in 3 mg L^−1^ MWCNTs/PVP consistently exhibited the highest total soluble protein contents in leaves compared to the other treatments. On day 9, the total protein content in water-treated flowers (control) was 6.57 g kg^−1^, and soluble protein content decreased by 52% from the initial level. On day 11, the protein content of leaves from cut stems kept in 6 and 9 mg L^−1^ MWCNTs/PVP decreased by 35% and 43%, respectively, relative to the initial level.

**Table 5:** Total soluble protein content in leaf of cut *Alstroemeria* flowers pulsed for 24 h with multi-walled carbon nanotubes (MWCNTs)/polyvinylpyrrolidone (PVP) composite during the vase period.

MWCNTs/PVP (mg L^−1^)	Total soluble protein (g kg^−1^)					
	Time (d)					
	1	3	5	7	9	11
0 (Control)	13.74 ± 0.6 a–d	11.63 ± 0.7 e–h	10.57 ± 0.7 hij	8.63 ± 1.1 k	6.57 ± 0.4 l	0.0 m
3	15.02 ± 0.4 a	13.57 ± 0.2 a–d	12.35 ± 0.6 c–f	12.90 ± 0.6 b–e	12.46 ± 0.3 b–f	11.17 ± 0.6 ghi
6	13.96 ± 0.2 abc	12.30 ± 0.9 def	11.24 ± 0.4 f–i	10.57 ± 0.2 ghi	10.29 ± 0.4 hi	9.12 ± 0.5 k
9	14.41 ± 0.5 ab	12.18 ± 0.5 efg	11.18 ± 0.2 f–i	10.35 ± 0.2 hi	9.63 ± 0.4 ijk	8.23 ± 0.4 k
F-test probability	< 0.0001					
LSD 0.05 (n = 3)	1.62					

The mean values followed by the same superscript letter are not significantly different at *P* ≤ 0.05, using LSD test. Values are means ± SE of three replicates.

### POX enzyme activity

The POX enzyme activity was increased in MWCNTs/PVP treated flowers compared to untreated cut *Alstroemeria*. Conversely, no change in the activity of the POX enzyme was observed during the vase life of control cut flowers (Table 6). However, it subsequently decreased in water-treated flowers on day 9 to 0.42 mmol min^−1^ kg^−1^.

**Table 6:** POX enzyme activity in leaf of cut *Alstroemeria* flowers pulsed for 24 h with multi-walled carbon nanotubes (MWCNTs)/polyvinylpyrrolidone (PVP) composite during the vase period.

MWCNTs/PVP (mg L^−1^)	POX activity (mmol min^−1^ kg^−1^)					
	Time (d)					
	1	3	5	7	9	11
0 (Control)	0.52 ± 0.01 jkl	0.44 ± .1 kl	0.52 ± 0.1 g–l	0.58 ± 0.01 hij	0.42 ± 0.01 m	0.0 n
3	0.68 ± 0.05 gh	1.03 ± 0.01 cd	1.10 ± 0.0 5c	1.23 ± 0.03 b	1.33 ± 0.03 b	1.48 ± 0.06 a
6	0.30 ± 0.01 m	0.58 ± 0.02 hij	0.68 ± 0.03 gh	0.77 ± 0.06 efg	0.90 ± 0.05 de	1.03 ± 0.03 c
9	0.59 ± 0.009 hij	0.55 ± 0.03 ijk	0.67 ± 0.03 ghi	0.75 ± 0.02 fg	0.87 ± 0.03 ef	1 ± 0.02 cd
F-test probability	< 0.0001					
LSD 0.05 (n = 3)	0.12					

The mean values followed by the same superscript letter are not significantly different at *P* ≤ 0.05, using LSD test. Values are means ± SE of three replicates.

### Malondialdehyde (MDA)

MDA accumulation increased exponentially over the vase life of water-treated cut *Alstroemeria*, reaching a peak of 17.33 µmol kg^−1^ on day 9. MDA levels gradually increased in flowers pulsed with MWCNTs/PVP (3 mg L^−1^) and peaked on day 11 (Table 7). Flowers treated with 6 or 9 MWCNTs/PVP reduced MDA accumulation compared to the control; the effect was more pronounced with the 6 mg L^−1^ concentration than with the 9 mg L^−1^ concentration.

**Table 7:** Malondialdehyde (MDA) content in leaf of cut *Alstroemeria* flowers pulsed for 24 h with multi-walled carbon nanotubes (MWCNTs)/polyvinylpyrrolidone (PVP) composite during the vase period.

MWCNTs/PVP (mg L^−1^)	MDA (µmol kg^−1^)					
	Time (d)					
	1	3	5	7	9	11
0 (Control)	4.97 ± 0.5j kl	7.13 ± 0.6 f.–i	10.33 ± 0.3 cd	15 ± 0.5 b	17.33 ± 0.3 a	0.0 m
3	3.31 ± 0.6 l	4 ± 0.04 kl	5.55 ± 0.5 ijk	7.25 ± 0.3 fgh	6.75 ± 0.4 ghi	7.17 ± 0.4 f.–i
6	3.50 ± 0.1 l	6.05 ± 0.9 h–j	8.32 ± 0.4 e–g	7.9 ± 0.7 e–g	9.01 ± 0.7 de	8.67 ± 0.6 def
9	4.77 ± 0.1 jkl	6.15 ± 0.6 hij	7.12 ± 1.03 f.–i	7.84 ± 0.7 e–g	11.7 ± 0.5 c	11.5 ± 0.8 c
F-test probability	< 0.0001					
LSD 0.05 (n = 3)	1.6					

The mean values followed by the same superscript letter are not significantly different at *P* ≤ 0.05, using LSD test. Values are means ± SE of three replicates.

### Stem end bacterial population

**Figure 3:**
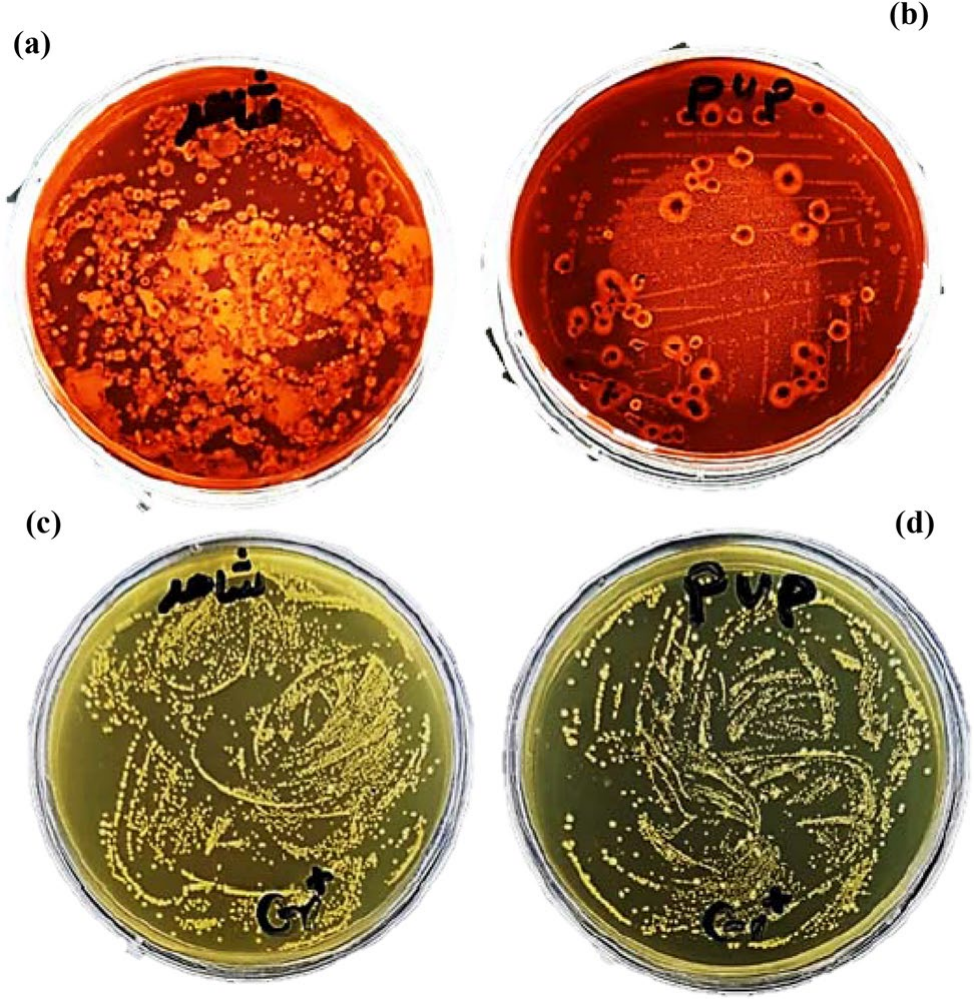
Representative bacterial culture plate images of the stem end of *Alstroemeria*. (**a**) Control (distilled water) in EMB (Eosin Methylene Blue) agar culture media as gram-negative bacteria. (**b**) multi-walled carbon nanotubes (MWCNTs)/polyvinylpyrrolidone (PVP) composite treatment (3 mg L^−1^) in EMB agar culture media as gram-negative bacteria. (**c**) Control (distilled water) in Azide Blood Agar Base culture media. (**d**) MWCNTs/PVP (3 mg L^−1^) in Azide Blood Agar Base culture media as gram-positive bacteria.

The gram-negative bacterial population was increased in the stem ends of untreated cut *Alstroemeria* flowers; the greatest increase was observed in control flowers (Table 8). Compared to the control, MWCNTs/PVP (3 mg L^−1^) treatment reduced the population of bacteria (Fig. 3). The highest reduction in gram-negative bacteria was observed with 3 mg L^−1^ MWCNTs/PVP, with 98.4% of bacteria exhibiting a low count (8 × 10^4^ CFU mL^−1^). Additionally, 3 mg L^−1^ MWCNTs/PVP demonstrated the highest reduction percentage in the gram-positive bacterial population at 91%, as the stem end had a bacterial count of 9 × 10^5^ CFU mL^−1^. Overall, the effect of nanocomposite on gram-negative bacteria inhibition was greater.

**Table 8:** Bacterial population of the stem end of cut *Alstroemeria* flowers pulsed for 24 h with multi-walled carbon nanotubes (MWCNTs)/polyvinylpyrrolidone (PVP) composite at end of the vase period.

Treatment	Gram-negative	Reduction (%)	Gram-positive	Reduction (%)
Control	5 × 10^6^ CFU mL^−1^	No reduction	1 × 10^7^ CFU mL^−1^	No reduction
MWCNTs/PVP	8 × 10^4^ CFU mL^−1^	98.4%	9 × 10^5^ CFU mL^−1^	91%

### Scanning electron microscopy (SEM)

**Figure 4:**
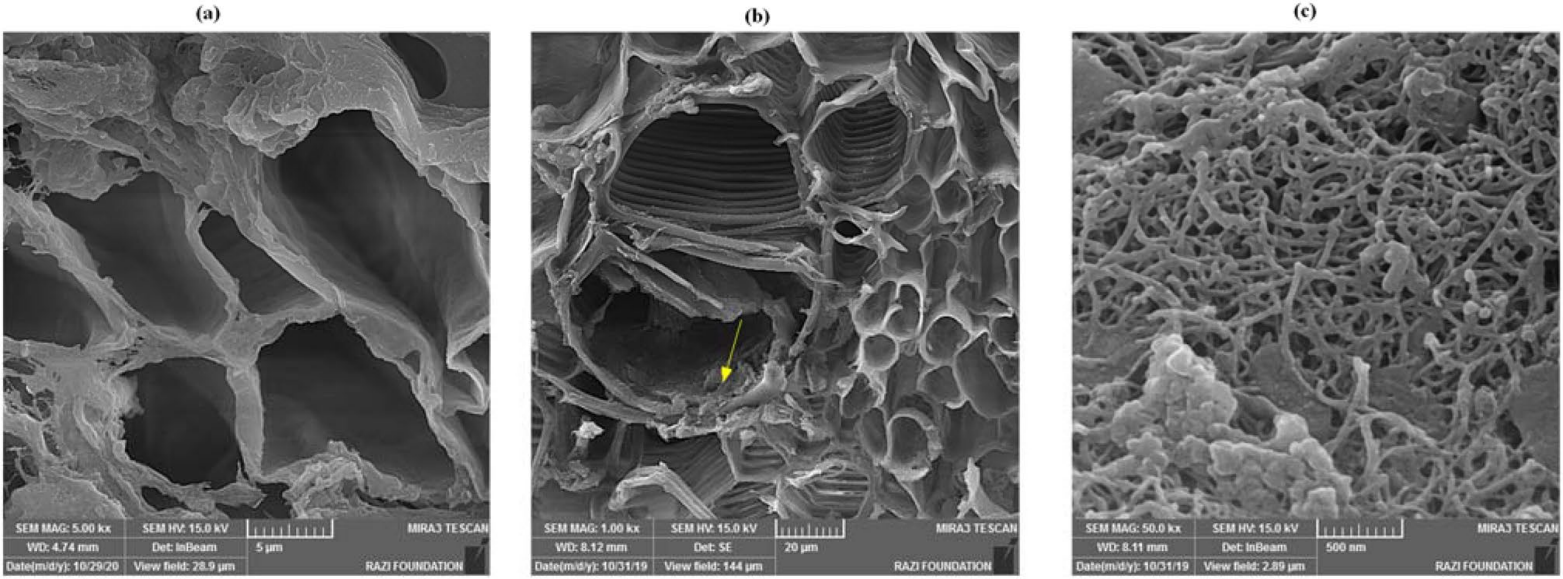
SEM (scanning electron microscope) images of inside of *Alstroemeria* stem with multi-walled carbon nanotubes (MWCNTs)/polyvinylpyrrolidone (PVP) showing their entry into the stem. (**a**) SEM image of the inside of *Alstroemeria* stem in control. (**b**) SEM image of inside of *Alstroemeria* stem with MWCNTs/PVP at low magnification (scale bar 20 µm), and (**c**) at high magnification (scale bar 500 nm). The yellow arrows represent aggregates of MWCNTs/PVP.

SEM analysis revealed that after the evaluation period, MWCNTs/PVP was detected in the stem of cut *Alstroemeria* and deposited on the stem's internal surface (Fig. 4). When applied in a vase solution, the images revealed that the MWCNTs/PVP could be allocated to stems and was easily visible within the stem. Therefore, this MWCNTs/PVP composite may serve as an effective scaffold for water movement in cut flower stems. In this study, we have demonstrated that the composite of MWCNTs and PVP possesses outstanding dispersion properties in aqueous media, such as vase solution.

## Discussion

Carbon nanotubes are well-dispersed in aqueous media and do not aggregate^49^. Given that the MWCNTs are delivered to the plant's stem via water, ensuring they are evenly distributed throughout the vase solution was critical. As previously described in the “Materials and methods” section, this was facilitated by the MWCNTs/PVP composite, and it was demonstrated (via TGA, X-ray diffraction, FTIR spectra, and TEM) that the MWCNTs/PVP composite exhibits excellent dispersion properties in vase solution.

The results indicated that MWCNTs/PVP could have a concentration-dependent effect on the vase life. MWCNTs/PVP at appropriate concentrations (3 mg L^−1^) prolong the life and delay the abscission of *Alstroemeria* florets. Our findings are consistent with recent reports demonstrating the beneficial effects of MWCNT and graphene oxide on rose^34,50^. However, at a high concentration of MWCNTs/PVP (9 mg L^−1^), several black spots in the cut stem end demonstrated that the MWCNTs aggregated within the xylem, which could potentially have detrimental effects such as inhibiting nutrient and water transport. This method confirms that the effect of MWCNTs/PVP on cut flower longevity was dose-dependent within a narrow dose range. Our findings, similar to those in carnation^51^, indicate that SWCNT has a toxic effect on the vase life of carnation at higher concentrations. Di Zhang et al.^51^ explicated that SWCNT cannot be absorbed or transported by plant vascular tissue and can therefore cause vascular tissue blockage.

Moreover, we demonstrated for the first time that MWCNTs/PVP delayed the yellowing of cut *Alstroemeria* leaves. According to Table 4, the total chlorophyll concentration in the leaves of cut stems treated with the MWCNTs/PVP solution was higher than in the leaves of control plants. Photosynthesis requires the chloroplast structure to be stable^52^. One possible explanation for this observed effect could be that MWCNTs are capable of penetrating the chloroplast, exhibiting the MWCNTs' ability to stabilize the chloroplast structure^53^.

Although little is known about the interaction of CNTs with plants, some mechanisms underlying the effect of CNT on plants are known. In line with our findings, Hu et al.^52^ observed that MWCNTs could increase the chlorophyll content and promote *Zea mays* growth by regulating carbon and nitrogen metabolism. CNTs travel through the symplastic and apoplastic pathways^54^ until they reach the mesophyll cells, where they modify the chloroplast absorption profile by stimulating light absorption in the ultraviolet, green, and near-infrared ranges of the spectrum, thereby increasing the chlorophyll content^6^. Additionally, electron transfer between carbon nanotubes and thylakoids has been reported^55^. One mechanism for the beneficial effects of MWCNTs has been their ability to promote the expression of genes involved in photosynthesis, thereby enhancing photosynthetic efficiency^56^. In addition, the increase in total chlorophyll content observed at low doses of MWCNTs/PVP (Table 4) may result from the increased photosynthetic activity^57,58^. Velikova et al.^59^ observed that SWCNTs could alter pea plants' leaf micromorphology and chloroplast ultrastructure, thereby affecting their photosynthetic activity.

Cut flower dehydration is a fundamental impediment to preserving their quality. Maintaining a balanced water supply and limiting transpiration and water loss from whole-cut flowers contribute to improving the postharvest quality of flowers^60^. Our findings suggest that MWCNTs can increase water uptake, resulting in increased fresh weight in the cut stem. MWCNTs have previously been shown to penetrate seed coats, promote germination and growth, and improve plant water uptake^22^. Furthermore, it can aid in advancing plant anatomy and physiology^20,61^. CNTs may facilitate water and nutrient uptake by increasing the size of the xylem cell and enhancing the vascular region.

Previous studies suggest that CNTs create new pores for water permeation and enhance capillary action. CNTs stimulate the conduction processes in the stem by forming a network of capillaries across the vascular tissue (xylem and phloem cells)^20^. Also, CNTs are transported within the vascular tissue via transpiration through leaves^62^. According to Lahiani et al.^63^, MWCNTs stimulate the expression of water channel genes (Aquaporins). One possible explanation for the enhancement of water uptake is that CNTs' passive transport is facilitated by their size, charge characteristics (predominantly negative), and interactions with lipids^62,64^. Following wounding in cut flower stem, the wound-induced responses activate and deposition of polyphenolic compounds, including lignin (which causes vascular occlusion), at the site of the wound occurred^65,66^.

Some studies have shown that the application of PVP in a culture medium cause elimination of phenolic compounds and reduces the browning of explants^67,68^. Because PVP can adsorb polyphenols, it can be argued that PVP, as a polyphenol absorbing agent, is effective in reducing vascular occlusion and has positive synergistic effects with MWCNTs in improving water uptake by the stem.

The MWCNTs used in this study increased the soluble protein content and the peroxidase enzyme activity (Tables 5 and 6). On the other hand, with increasing MWCNTs concentration (6 and 9 mg L^−1^), biochemical activities failed to increase. This shows that the concentration of 3 mg L^−1^ is optimal for increasing *Alstroemeria*'s postharvest quality. It is evident that nanotubes should be used in a certain range for the desired effect. As a result, excessive exposure to MWCNTs/PVP may be toxic to the plant's biochemical and physiological processes. Exposure to MWCNTs at higher concentrations could damage various metabolic activities, consequently leading to the accelerating senescence of plants. In this study, as expected, the application of MWCNTs/PVP in vase solution led to a low level of MDA in leaves (Table 7), which may result from the material's ability to prevent lipid peroxidation.

Similarly, Ren et al.^69^ found that single-wall carbon nanotubes increased antioxidant system activity while lowering MDA levels. Zhao et al.^53^ demonstrated that exogenous MWCNTs maintain a low level of reactive oxygen species (ROS) and regulate the expression and activity of enzymes involved in the ASA-GSH cycle^53^, increased antioxidant activity by removing reactive oxygen species, resulting in increased protein stability, decreased lipid peroxidation, increased membrane integrity, and improved plant quality^69,70^.

CNTs can extend the vase life of cut flowers as a result of their high antimicrobial activity. MWCNTs/PVP reduced the bacterial population (particularly gram-negative bacteria) at the cut stem end in this study. Other studies have observed comparable results. Chen et al.^71^ demonstrated that MWCNTs exhibit inhibitory activity against various microorganisms. Numerous factors affect the antimicrobial properties of CNTs. Modified CNTs may demonstrate potent antimicrobial and anti-adhesive properties^17^. Other than the type of CNT nanocomposites used, the most important factors influencing the antimicrobial efficacy of CNTs are mechanical damage caused by cell disruption, the release of intracellular content, and the generation of oxidative stress.^17,72^. Our findings corroborated with Fang et al.^73^, who demonstrated that reducing bacterial blockages in the xylem vessels improves the water uptake and vase life of cut flowers. Also, Cai et al.^67^ reported that adding PVP to the culture medium of petal explants of *Paeonia lactiflora* was an effective method of inhibiting contamination. Due to their antimicrobial properties, MWCNTs/PVP composites have great potential for use in postharvest cut flowers.

## Conclusion

In conclusion, a solution containing a well-dispersed MWCNTs/PVP composite is a novel method for facilitating plants' uptake of this nanotube. The present findings unequivocally demonstrate that MWCNTs delay leaf yellowing by affecting water absorption, chlorophyll content, and antimicrobial properties, all of which are critical for the longevity of cut flowers. Unlike control plants, where leaf yellowing occurred abruptly on the cut stem, nanocomposite-treated plants gradually lost their green color from their base upward. Our research sheds new light on the interaction between MWCNTs, and *Alstroemeria* cut flowers and establishes the rationale for MWCNT application postharvest. This study represents a reliable advancement in increased water uptake in cut stems, resulting in beneficial physiological responses because cut flowers retain their ornamental value and quality. Moreover, additional research on MWCNTs' interactions with cut flower systems is necessary to elucidate their longevity and toxicity mechanisms.

## Materials and methods

### Plant material

The present investigation involving plant materials adheres to all applicable institutional, national, and international guidelines. In addition, the study does not utilize particular plant materials from the experimental area. The plant materials were identified using a catalog available at the greenhouse located in Ashianehsabz, Tehran, Iran. The experimental material consisted of freshly cut, commercial-grade Alstroemeria (*Alstroemeria hybrida*) 'Sarah' flowers. Fresh flowers were harvested in the morning when the first florets began to open, and others had developed 50% color (commercial stage). Cut stems were immediately transferred to the laboratory of Ilam University and trimmed to a length of 40 cm under deionized water before treatment to avoid stem air blockages. Afterward, lower leaves were stripped from cut stems and placed in vase solutions. Each cut stem was placed individually in the vase containing the vase solution. Finally, all cut stems were kept in a vase life room with a controlled temperature of 20 ± 2 °C, 65 ± 5% relative humidity, and a light intensity of μmol m^−2^ s^−1^ under a daily photoperiod of 12 h.

### Composite preparation and characterization

PVP25 with an average molecular weight of 25,000 (Merck) was used in this study, and MWCNTs (Purity > 95%, OD 8–15 nm, L 5 µm) were purchased from Neutrino Company in Iran. An ultrasonic bath was used to coat MWCNTs with a PVP polymeric surfactant in an aqueous solution. First, 150 mg of MWCNTs were mixed with 200 mL of deionized water before being sonicated (40 kHz) for 15 min. Approximately 65 mg of PVP was added to the MWCNTs water solution, mixed, and placed in an ultrasonic bath (40 kHz) for 3 h to obtain PVP-wrapped MWCNTs solution^74^. Fourier transform infrared spectroscopy (FTIR), thermogravimetric analysis (TGA), X-ray diffraction (XRD), and transmission electron microscopy (TEM) were used to confirm the PVP coating of MWCNTs. Figure 1a–f depict the data.

### Pulsing treatments

The nanocomposite concentrations were determined using the results of pilot experiments. Cut stems were conditioned in MWCNTs/PVP composite pulse solution at 0, 3, 6, and 9 mg L^−1^ for 24 h and then transferred to deionized water in vases. Cut *Alstroemeria* stems were kept in 250 mL glass vases (Erlenmeyer flask) containing 100 mL of deionized water as vase solutions. The vase solutions were changed daily. The mouths of the vases were covered with aluminum foil paper to prevent contamination and reduce evaporation.

### Evaluation of vase life, floret abscission, and leaf yellowing

The vase life of cut *Alstroemeria* flowers was determined by the length of time between the first day the flowers were placed in the vase solution and the last day when half of the florets or leaves had wilted, discolored, or abscised^75^. The first abscised floret of individual inflorescences was recorded to determine the timing of floret abscission during vase life. Leaf yellowing was defined as the number of days required to discolor 50% of the leaves on a cut stem^75^.

### Measurement of relative fresh weight and water uptake

Throughout the vase life of each replicated cut flower, the daily relative fresh weight (RFW) was determined using an analytical balance and expressed as a percentage of the initial fresh weight (FW) on day 0^76^. Daily changes in water uptake (L kg^−1^) of the cut stems were calculated from volume losses in the vase solutions and expressed as the difference between water uptake and fresh weight change^76^.

### Chlorophyll content determination

Chlorophyll content in *Alstroemeria* leaves was determined through the Lichtenthaler and Buschmann^77^ method. Absorbance was measured using a spectrophotometer (Analytik Jena AG, Specord 50 Plus, Germany) at 663 nm and 647 nm. Total chlorophyll content was expressed as g per kg on a fresh weight basis (g kg^−1^).

### Determination of leaf soluble protein content, POX enzyme activity, and malondialdehyde (MDA)

Initially, 0.2 g of frozen leaf tissue was ground in liquid nitrogen using a mortar and pestle to determine enzyme activity. The POX activity (EC 1.11.1.7) was determined by measuring the increase in absorbance at 470 nm every 15 s for 1 min^78^. The enzyme activity was expressed in mmol within 1 min per kg fresh weight basis (mmol min^−1^ kg^−1^). The protein concentration was measured using the Bradford^79^ method. The absorbance was measured using bovine serum albumin (BSA) as a standard at 595 nm. Total protein content was expressed as g per kg on a fresh weight basis (g kg^−1^). The MDA content was estimated according to Heath and Packer^80^. Afterward, the MDA content was determined via a spectrophotometer at 532 and 600 nm and expressed as μmol per kg on a fresh weight basis (µmol kg^−1^).

### Measurement of stem end bacterial population

After the experiment, the 2-cm-long cut stem end of *Alstroemeria* was crushed and homogenized, then diluted with water. The extract was then divided into aliquots (0.1 mL) and spread on nutrient agar plates. An incubator programmed to 37 °C was used to maintain the Petri dishes for 48 h. Gram-negative bacteria were cultured on Eosin Methylene Blue (EMB) agar, while gram-positive bacteria were cultured on Azide Blood Agar Base. Following that, log CFU mL^−1^ counts of gram-positive and gram-negative bacteria were performed^81^.

### Scanning electron microscopy (SEM) observation of the stem section

The distribution of MWCNTs in the cut stem was performed using FE-SEM (MIRA3, TESCAN). Preparation of the samples for imaging was determined according to the Haberman et al.^82^ method with different steps of fixation, saturation, and drying. Briefly, stem tissues were fixed in 0.1 M phosphate buffer (pH 7.2) containing 5% glutaraldehyde for 24 h, followed by five washes in phosphate buffer. The stem tissues were gradually saturated with increasing concentrations of ethanol (25%-100%). Stem tissues were then dried in a critical point dryer device for about 2 h.

### Statistical analysis

ANOVA was used to evaluate the results statistically using SAS 9.4. The LSD multiple range tests (*P* ≤ 0.05) were used to determine any statistically significant differences between the treatment means. Notably, the mean comparison between treatments was performed until the end of the vase life of the control treatment. All values were expressed as the means ± standard errors of the means.

## Data Availability

The datasets analyzed during the current study are available from the corresponding author on reasonable request.
